# Nurses’ collegiality: An evolutionary concept analysis

**DOI:** 10.1177/09697330231221197

**Published:** 2023-12-18

**Authors:** Mari Kangasniemi, Sunna Rannikko, Helena Leino-Kilpi

**Affiliations:** 8058 University of Turku; 8058 University of Turku; Turku University Hospital; 8058 University of Turku

**Keywords:** Collegiality, concept analysis, ethics, intra-professional, nurse, Rodger’s concept analysis

## Abstract

Collegiality is one of the fundamental values of the nursing profession. During the nursing history, collegiality has been described as part of a nurse’s relationship with their peers and it influences the quality of care they provide and job satisfaction and commitment to their work. Despite earlier definitions, the concept of collegiality in nursing has remained unclear. The aim of this study was to clarify the concept of collegiality in the nursing profession, using Rodger’s evolutionary concept analysis. We carried out electronic searches using the CINAHL, PubMed, Scopus, Web of Science, SocINDEX, PsycINFO and Eric databases and manual searches of the reference lists of the selected papers. The searches were limited to peer-reviewed papers published in English language from the inception of database to November 2022. This identified 25 papers. Based on our analysis, the attributes of the concept of collegiality were achieving mutual goals together with equality, reciprocity, trusted advocacy, powerful self-regulation and engaged belongingness. Antecedents of the concept included existing professional group, connection between professionals and professional self-esteem. The consequences were strengthening nurses’ professional status, job satisfaction and their ability to provide the best possible patient care. We found that nurses’ collegiality was a value-based concept, with a unique character based on professional connections. The concept brought together ethical and pragmatic strategies to achieve the best possible results for the nursing profession. Provided knowledge can be applied for further development of the concept and applying it in clinical research and practice. The concept of nurses’ collegiality should also be studied in the future because both the profession and their working environment are constantly changing.

## Introduction

Nursing is based on shared professional values, which encompasses autonomous and collaborative care and promotes the health of individuals and groups of all ages and during all phases of their lives.^1,2^ Nurses’ peer-relationships with their colleagues have been seen as an inherent part of nursing.^3–5^ They work together to achieve the goals of their profession and ensure that patients receive high-quality, safe and seamless care.^6,7^ Peer relationships tend to be based on collegial professionalism,^3^ where groups of nurses collaborate,^7–9^ share knowledge and support their peers.^10–13^

The value that collegiality has for nursing profession^14,15^ is topical and crucial for several reasons. Current healthcare services are complex and collegiality is required when patient care is being developed and delivered^16^ and collegiality has found to enhance improved patient outcomes.^17^ Collegiality has seen as a part of ethical basis of profession,^15^ strengthening the unity of the profession,^8^ supporting intra-professional collaboration between generations^15^ and between nurses in different healthcare specialities^18^ and roles.^19^ In addition, healthcare has faced a number of crises, such as staff shortages^2,20^ and the COVID-19 pandemic.^21,22^ Also increasingly common remote work has changed the nature of work, and work relationships added the discussion about collegiality.^23^ Previous empirical results have highlighted the need for conceptual knowledge^14^ to support nurses’ collegiality^5–7,24^ and to identify possible barriers to it.^25^ Although this subject is topical, and interest in researching this area has increased, there is still need to clarify the concept of collegiality in nursing.

## Background

Collegiality refers to relationship between individuals in professions or workplaces,^26,27^ who strive to achieve a common purpose.^26,28^ Dictionaries define the terms *collegiality* and *collegial* as adjectives and synonyms for collegiate. The origin of the noun *collegiality* has been defined in the late Middle Ages in English, the Latin word *collegialis* means partnership and the French use the word *collégialité*.^29^ The roots of collegiality have been placed on the Hippocrates’ work on medical collegiality,^30^ but the term collegiality has been located on the early Christian denominations, where bishops were seen to have shared responsibilities with ancient Apostles to serve people.^31^ Later on, Max Weber interprets collegiality negatively, as a control means of autocrats to prevent experts from challenging organizational power.^30,32^ In 19th and early 20th century sociology, collegial structures were viewed as forums within which communication could take place between those in highly specialized roles, to ensure preservation of shared ethical standards to mitigate the self-interest of individuals but also to arbitrary exercise in power.^32^ Various dictionaries define it as cooperative interaction,^33^ a friendly relationship between people who work together or do the same job^34^ and have shared responsibilities^29,35^ or authority.^35^ The term *colleague* is defined as an associate, fellow worker or co-worker, who is connected by their profession and has a similar rank or status.^36^ However, the definitions do not specify what kind of interaction colleagues have during collegiality. In addition, there have been no distinctions among the different terms referring to colleagues.^37^

As a concept, collegiality involves both descriptive and normative elements. As a description, it provides information about what qualifies two or more people to be called colleagues.^37^ Colleagues have been described as individuals who know each other,^38^ do the same work, work in the same area at the same institution, have a common purpose and have the same status or level of responsibility.^37^ According to Reuter et al. (2020),^38^ a colleague is a dual concept as it can include both positive and negative characteristics. In contrast, the concept of collegiality has been described as normative as it explains the potential value and normativity of relationships between colleagues.^37^ These relationships consist of a number of aspects. Cultural aspects refer to sets of beliefs that group members share. Structural aspects refer to a systematic set of decision-making rules that govern the group. Behavioural aspects refer to the roles and interactions of the members.^28^

Nurses’ collegiality has been defined as a unique condition among members of a formally organized professional working group,^39^ and it comprises supportive collaboration and satisfaction with, and respect for, each other.^11^ It has been seen as a positive,^11^ non-hierarchical relationship between nurses in different roles,^13^ where work-related and social exchanges are used to make decisions and put them into practice.^39^ Nurses’ collegiality has been described as part of professionalism^40^ and as a form of self-regulating the nursing profession.^6^ The roots of nurses’ peer-relationships have been found in sisterhood, where nurses were connected by their social positions and professional identities, based on an ethical or religious-based vocation.^41^ Thus, nurses’ collegiality seems to have a value-based structure^3^ and ethical nature, which refers to the golden rule of people focussing on supporting their colleagues rather than themselves.^42^ As an expression of existence of collegiality, nurses’ collegiality guidelines^15^ have been developed and collegiality has been included in the ethical codes of nursing. Based on codes, nurses collegial respect and confidentiality^2,43–45^ have been described as a professional expectation that must be displayed.

Nurses^46,47^ and nursing educators^14,48^ have perceived that collegial support promotes their retention at work and has had a positive association with burnout.^47^ It has improved nurses’ job motivation,^11,49^ encouragement in work^46^ and commitment to their organization.^50^ Nurses’ collegial solidarity has found to have positive effect on organizational climate,^50^ and collegiality has supported nurse academics to manage challenges or negative outcomes at work.^13,51^ Lack of collegiality has reported to be a predictor for missing or neglecting to provide nursing care,^52^ and midwives have experienced that it contributes to better births.^17^ They have reported that collegial support enabled care when it was experienced as positive, sharing knowledge and responsibility, but prevent care when it was experienced as a compensate for each other’s weaknesses and did not support midwives pride in being part of a professional group.^8^ In addition, nurses’ have also found to have poor collegial relationships.^14^

In the nursing literature, collegiality not only included peer relationships between nurses in different roles^17,23^ in clinical practice^4,6,7,10,11,15^ and research^13^ but also covered relationships with other colleagues. For example, it has been used to describe nurses’ inter-professional collaborations with physicians^25,53,54^ and nursing educators^55^ and those working in the social care sector.^48^ The aim of inter-professional collegiality has been to promote communication, facilitate decision-making,^52,55,56^ achieve mutual learning, share knowledge sharing^48,56^ and enable collegial mentoring.^25,48,56^ It has been seen as a strategy to support a united and safe working culture^48^ and ethics among professionals.^57^ Collegiality has also been connected to work environment cultures, where the combined power of all the staff members improved patient care.^26,53^ Collegiality has also been studied as part of nurse educators’ professional relationship,^14,48,55^ where friendship-based, honest and open communication resulted in positive outcomes for both individuals.^55^ It has also been reported that collegiality created unique relationships and opportunities for nurses to network and create business ventures.^58^ It also supported to develop relationships with informal caregivers in healthcare, such as doulas who support women during and after childbirth.^59^

## Aim

The aim of this study was to clarify the concept of collegiality in the nursing profession by using the concept analysis method developed by Rodgers.^60^ We wanted to produce knowledge that provides understanding of collegiality and way to apply the concept in nursing research and clinical practice. The research question was: what are the attributes, antecedences and consequences as well as surrogate and related terms of the concept of nurses’ collegiality?

## Methods

We used Rodger’s evolutionary concept analysis method^60^ to clarify the concept of collegiality in nursing. Rodgers’ approach is dispositional, rooted in the structuralist and hermeneutic schools, where concepts are dynamic, changing and contextual. They are abstractions that are expressed in words, and words are the mental cluster that lies behind the word. Examination of the common use of a concept provides a means to explore the underlying concept and to identify its definition. For Rodgers, the process of concept development is a cycle that implies the application, significance and use of a concept. This method was particularly suitable for this study because we understand collegiality as a concept that develops and changes over time and across disciplines. Evolutionary concept analysis enables us to conduct an inductive clarification of the concept based on the use of the concept.

The evolutionary method of concept analysis consists of six, simultaneously performed steps, organized in three phases by Tofthagen and Fagerström.^61^ In the first, initial phase, concept is chosen for the analysis, identifying the context, collecting material for the analysis and choosing the papers for the analysis. On that phase, we chose the concept of collegiality in the context of nursing because there are no previous analyses. This has been described in the introduction section of this paper. In addition, we carried out literature searches to identify the use of the concept collegiality in the previous literature. The second phase was analysis, where based on chosen literature, we considered the antecedences, attributes and consequences of the concept and the surrogate and related terms of collegiality as a concept. That was reported on the result section. As third phase, we identified how research could be used to further develop the concept and reported this in the discussion section.^61^

### Literature searches

**Figure 1.**
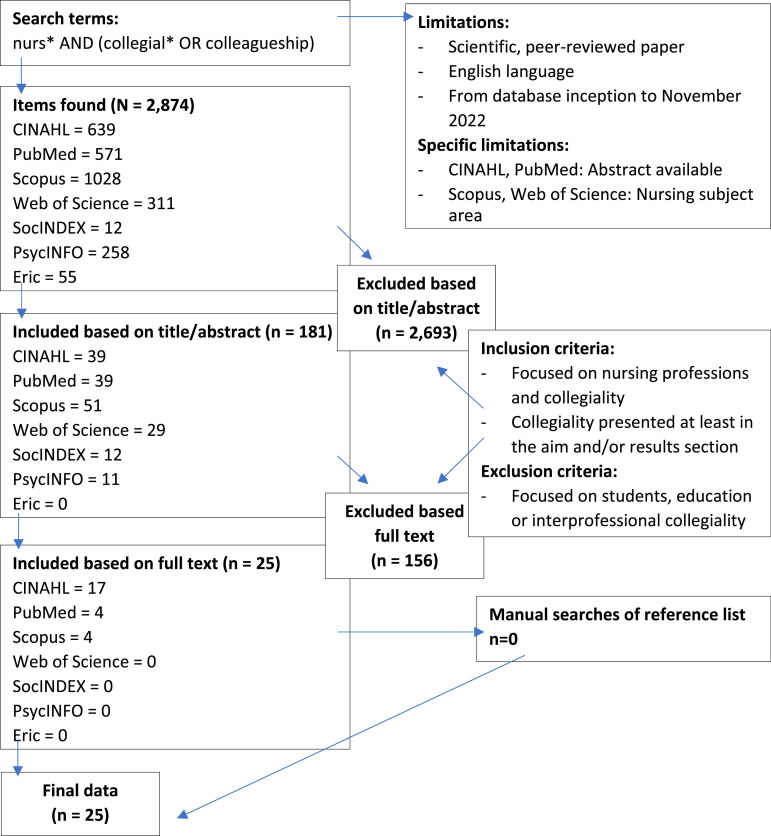
Flow chart of the literature searches and the number of papers that were selected.

We carried out both electronic and manual literature searches. The electronic searches were conducted using the CINAHL, PubMed, Scopus, Web of Science, SocINDEX, PsycINFO and Eric databases. The search terms were based on our preliminary literature searches, using dictionaries and consulting an informatician specialist. To ensure that the searches were comprehensive, we used free search terms related to *collegiality* and *nurses* and combined them with Boolean operator (Figure 1). The searches were limited to scientific, peer-reviewed papers that were published in the English language from the inception of the databases to November 2022. In addition, the CINAHL and PubMed searches were limited to papers that had an abstract available. The Scopus and Web of Sciences searches focused on the papers in the nursing subject area. We carried out manual searches of the reference lists of the selected papers using the same limitations and inclusion criteria.

We selected papers based on inclusion and exclusion criteria. Papers were included papers if they focused on various nursing and allied professions such as public health nurses, midwives and physiotherapists and used the term collegiality or related words, such as colleagueship or collegial. In addition, collegiality had to be presented at some point in the paper, at least in the aim and/or results. Studies that focused on students, education or inter-professional collegiality were excluded. The electronic searches identified 2874 papers and we selected 181, based on the title and abstract and the 25 based on the full text of those papers. The selection was carried out independently by two researchers (MK, SR).

### Data analysis

We analysed the data that responded to our research question. First, we read the data several times to get an overview of the entire data. This was supported by tabulating the data according to the author(s), years and country and the aims, methods and main results of the study (Table 1). After that, we coded the data thematically^60^ based on attributes, antecedents and consequences, together with relevant and alternate terminology and how the concept was used. To identify the attributes of the concept, we looked for all statements describing core characteristics of the concept. The antecedents were identified by asking what happens before and consequences, what happens after or as a result of the concept.^60^ We collected all expressions that described nurses’ collegiality, regardless of whether they produced empirical results, theoretical reflections or how many times they were referred to. We continually organized and reorganized corresponding points in the literature until a cohesive and comprehensive system of descriptions was generated. There were no previous concept analyses available or direct descriptions of attributes, antecedents, consequences or other parts of the concept analysis. Two researches (MK, SR) reflected on, and discussed, all expressions related to nurses’ collegiality and the organized according to the method. The analysis was finalized and produced as descriptive text, with input from all the all researchers.

**Table 1.** Selected papers for concept analysis.

Author(s), year, country	Aim	Methods
Hinshaw and Field, 1974, USA^61^	To investigate professional nurses, using Blau’s theory of esteem or status giving, which is a model of collegial evaluation	Quantitative study that tested the model on 117 registered nurses. Analysed with statistical methods
Nolan, 1976, USA^55^	To discuss the importance of colleagueship among nurses	Discussion paper with nine references
McMahon, 1990, UK^10^	To analyse power and collegial relations among nurses on wards with primary nursing and hierarchical management structures	Mixed-methods study that comprised observations on four wards and a survey that involved 45 nurses. Analysed with statistical methods
Cowan and Tveit, 1994, USA^64^	To develop a collegial model for clinical nurse specialists	Mixed-methods study with literature review and group discussions between clinical nurse specialists and chief nursing officers. Numbers not specified. Analysis method not specified
Geiger-Bronsky et al., 1994, USA^17^	To describe the implementation process of a collegial model for clinical nurse specialists	Description of the implementation process of a collegial model for clinical nurse specialist
Hansen, 1995, USA^33^	To implement a conceptual model for collegiality among acute care nurses	Mixed-method study using previous literature and interviews for professional nurses, to develop a model tested by 539 nurses. Statistical analysis
Dickerson et al., 2007, USA^57^	To give voice to the concerns of registered nurses (RNs) who wrote comments on a survey about RNs’ intent to work	Qualitative study based on written comments in a survey by 472 registered nurses. Analysed with thematic analysis
Rubin et al., 2009, USA^63^	To use a focus group to identify barriers to communication between nurses and nursing aides and find solutions to measure the impact of this focus group on job satisfaction	Mixed-method study, with a randomized pre-test/post-test control group study design. Individual interviews with two registered nurses, ten licensed practical nurses and nineteen nursing aides. Focus group discussions with 12 participants and a survey. Analysed using qualitative and statistical analysis
Cowin, 2013, Australia^11^	To explore the challenges, strengths and strategies used during nursing team communications in order to build relationships	Qualitative study with exploratory approach using focus groups for six registered nurses and thirty enrolled nurses. Analysed with constant comparison method
Miller and Kontos, 2013, Canada^7^	To examine neurorehabilitation nurses’ intra-professional and inter-professional practices, during an arts-based education intervention	Qualitative, secondary analysis, observations of structured and unstructured activities baseline, 3 and 12 months postintervention. Semi-structured interviews with 31 licensed practitioners. Modified directed content analysis approach used
Padgett, 2013, USA^13^	To understand how staff nurses managed variations in group practices and negotiated the rules used for quality of care, collegiality and accountability	Qualitative, critical ethnography study design. Participant observations with semi-structured interviews for nineteen nurses, four managers or assistant managers, two nursing educators and one ward clerk. Policy analysis
Broadbent and Moxham, 2014, Australia^58^	To better understanding the interdisciplinary relationships needed to sustain an effective emergency mental health triage service for mental illness patients in an emergency department	Qualitative, ethnography study design. Individual and group interviews and observations between seven mental health triage nurses and twenty-eight emergency nurses. Guidelines and protocols were analysed with a holistically oriented approach based on the constant comparative method
Padgett, 2015, USA^56^	To explore the practices of collegiality and conflict in nursing	Discussion paper, based on the experiences of a nurse drawn from a larger critical ethnography study. Narrative structure analysis
Stewart et al., 2015, New Zealand^59^	To gather nurses’ stories about early experiences of nursing, particularly collegiality	Qualitative study with oral history methodology. Focus group discussions for eight registered nurses and two enrolled nurses. Thematic analysis method
Noguchi-Watanabe et al., 2016, Japan^40^	To develop a conceptual framework on how perceived support from colleagues helped to retain registered nurses	Qualitative study based on grounded theory. Semi-structured interviews with 26 registered nurses and 23 participant observations. Analysed with a constant comparative method
Gustafsson et al., 2017, Sweden^8^	To deepen the knowledge about how midwives’ lived experiences of caring for new mothers with initial breastfeeding difficulties	Qualitative study with a reflective lifeworld approach. Interviews for six midwives. Analysed using phenomenological methods
Kangasniemi et al., 2017, Finland^15^	To develop nurses’ collegiality guidelines	Delphi study with systematic review and an online panel with 35 experts in the first and 40 experts on the second round
Madden et al., 2017, USA^60^	To offer an initial theoretical understanding of communication between nurses and certified nurse assistants	Qualitative, grounded theory approach. Observation, shadowing and semi-structured interviews with seven nurses and ten certified nurse assistants. Constant comparison method
Froneman et al., 2019, South Africa^22^	To explore how the professional dignity of hospital-based midwives could be enhanced, based on their experiences	Qualitative study with descriptive phenomenological research design, using in-depth interviews with 15 midwives, analysed using phenomenological method
Kılıç and Altuntaş, 2019, Turkey^41^	To examine the effect of an organizational climate to collegial solidarity among nurses	Quantitative study with 544 nurses. Colleague Solidarity of Nurses’ Scale and the Organizational Climate Scale used, statistical analysis
Göktepe et al., 2020, Turkey^39^	To evaluate how work-related variables and nurses’ collegial solidarity affect their job motivation	Quantitative study with 172 nurses. Colleague Solidarity Scale for Nurses and Nurses Job Motivation Scale used, statistical analysis
Halberg et al., 2020, Denmark^9^	To examine nurses’ experiences of job rotations following the patient's pathway in a hospital	Qualitative study with semi-structured focus group interviews with 16 nurses. Analysed with thematic analysis
Theodosius et al., 2020, UK^62^	To investigate the impact of emotional labour between nurses and patients/colleagues on emotional exhaustion, depersonalization, personal accomplishments and intention to leave	Quantitative study with 118 nurses. Cross-sectional descriptive design using the Emotional Labour Scale, the Maslach Burnout Inventory and intention to leave questions. Analysed with statistical methods
Laugensen et al., 2022, Denmark^49^	To explore how COVID-19 changed work environment and influenced nurses’ clinical decision-making	Qualitative study with three focus groups with fourteen nurses. Analysed with thematic content analysis
Ylitörmänen et al., 2023, Finland^12^	To describe registered nurses’ experiences of collaboration with other nurses in a Finnish and a Norwegian hospital	Qualitative study, interviews with 29 nurses. Analysed with inductive content analysis

### Description of the studies

We analysed 25 papers that met the inclusion criteria: 13 papers used qualitative methods, 4 used quantitative methods and 4 were mixed-method studies. There were also two discussion papers,^62,63^ one implementation process description^18^ and one Delphi study with qualitative and quantitative rounds.^15^ Interview methods were used to collect data in most of the qualitative studies.^7–9,11,12,24,46,56,63–67^ Written data^64^ and observations were also used.^7,65^ Surveys were used to carry out the quantitative studies.^47,49,50,68^ The mixed-method studies combined observations,^10^ surveys,^10^ pre-test and post-test measures,^69^ literature reviews^39,70^ and interview data.^39,69,70^ There were 2262 informants for the empirical studies, and the cohorts varied from 14 to 544 participants. Ten of the studies were conducted in the USA, two each in Australia, Denmark, Finland, Turkey and the United Kingdom and one each in Canada, Japan, New Zealand, South Africa and Sweden.

## Results

**Figure 2.**
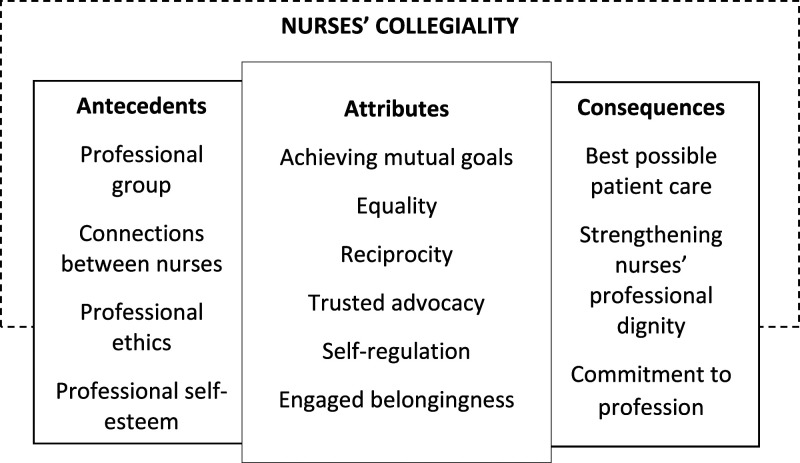
Attributes, antecedents and consequences of the concept of nurses’ collegiality.

Based on our findings, nursing colleague was a professional with the same education as another nurse but can be at a different stage of their career.^69^ Collegiality was a concept that created value-led basis for goal-oriented collaboration. Antecedents described the shared idea of profession and the consequences that the contributions that individuals and groups of professionals had on the quality of care (Figure 2).

### Attributes of the concept of nurses’ collegiality

According to Rodgers (2000),^60^attributes represent the real meaning of concepts, rather than nominal definitions, and provide an opportunity to identify the context of a concept. This analysis showed that the attributes for the concept of nurses’ collegiality were achieving mutual goals, equality, reciprocity, trusted advocacy, powerful self-regulation and engaged belongingness.

*Mutual goal achievement* was the attribute of collegiality that created a reason for two or more professionals to become colleagues and a purpose for the connection. The goals were the professional intellectual interests that nurses shared^62^ and what colleagues could achieve together.^10,15,39,49,62,63,70^ These goals were based on professional nursing values^15^ and were realized when the nurses provided the best possible patient care.^15^ To achieve mutual goals, individual nurses needed to be committed^39^ and ready to work as a team and share efforts,^10^ power^49^ and responsibility between colleagues.^8,15,49,56,70^ Strategies for mutual goal achievement^8^ required nurses to complement each other’s skills and capabilities, in order to enhance the benefits they provided patients.^8,24^ It involved mutual learning,^69^ including shared knowledge,^7–9^ information^63^ and exchanging ideas between colleagues^11^ during different phases of their career.^15,69^

*Equality* was the attribute that referred to colleagues having equal relationships as individuals and as part of a group.^15,24,39,70^ Equality was about respecting^11,15,18,24,49,70^ and treating colleagues as human beings^15^ at all stages of their career.^69^ It was a non-hierarchical relationship among nurses^15,39,49,70^ regardless of their rank.^62,70^ Equality drove how colleagues distributed their professional responsibilities at work.^8,15,56,70^

*Reciprocity* was the attribute of collegiality that referred to an unreserved and mutual exchange of professional elements between colleagues. It was expressed during communication, support and collaboration. Reciprocal communication between colleagues was open,^10,15^ dialogic, courteous^15^ and respectful.^11^ It emerged when colleagues were making and implementing decisions,^39,49,56^ during mutual consultations^62^ and when nurses provided feedback to each other.^12,46^ Reciprocity emerged during collegial support^10–12,18^ when nurses were planning,^12^ executing^10^ and optimizing patient care.^24^ It focused on working tasks^7,24,56,63^ and moral^63^ and emotional reflections.^7,24,46^ Reciprocity during collegial collaboration meant assisting each other.^12,46,63^

*Trusted advocacy* as an attribute in nurses’ collegiality^10,15,18^ was nurses feeling confident that individual and groups of colleagues^18^ would stand by them. It was about nurses feeling they would receive protection, deference^63^ and advocacy^15^ from colleagues during their daily work^46,63^ of if they risked making a mistake.^46^ It also referred to colleagues intervening if they felt things were going wrong.^15^

*Self-regulation* was the attribute that referred to nurses’ autonomy^15,24^ and integrity^49^ to monitor and follow-up what is happening in their profession.^46^ It referred to nurses sharing power^10,49,63,70^ in order to achieve^11,64^ and protect their professional goals^46^ without external interference.^18,70^

*Engaged belongingness*^9,11,31^ was related to a sense of community^64^ and cohesiveness.^18,39,49,64^ It was related to nurses’ voluntary and positive^64^ commitment,^39,56^ accountability^39^ and how colleagues depended on each other^63^ and was demonstrated by friendliness^10^ and valuing each other’s opinions.^11^ Engaged belongingness resulted in solidarity^64^ and created strong bonds with colleagues.^24^

### Antecedents of the concept of nurses’ collegiality

Antecedents refer to the events or conditions that must exist before a concept is possible.^60^ Based on our analysis, the antecedents of nurses’ collegiality were an existing professional group, connections between professionals in that group, shared professional ethics and professional self-esteem. *Professional group* meant a definable group of members organized by their profession.^7,10,18,24,39,49,62^ Formally organized groups^49,65^ shared the same intellectual interests,^65^ such as scientific research,^62^ educational background^65,66,68^ and field of expertise.^68^ Education provided the basis for knowledge and normative aspects of nursing.^68^

One antecedent for nurses’ collegiality was a *connection between* two or more people.^10,24,46^ They may have worked together,^10,63,65^ but this did not have to be in the same physical workplace, as long as they had the same work focus or interest.^65^

The profession needs to have shared values based on *professional ethics*.^15^ Shared values unify the profession and create professional norms^68^ and standards for their daily work.^15,63,68^ Values are also expressed in nurses’ professionalism.^63^

*Professional self-esteem* as an antecedent for nurses’ collegiality meant acknowledging the profession^8,39,68^ and professionals,^15,68^ regardless of their professional merits.^15,39,49,70^ Professional self-esteem required that nurse peers are to be seen, esteemed^68^ and to respect each other.^11,15,49^

### Consequences of the concept of nurses’ collegiality

The consequences of a concept refer to what happens when the concept has been used.^61^ In case of nurses’ collegiality, the identified consequences were providing the best possible patient care, strengthening the nurses’ professional dignity and their commitment to the profession. Studies have shown that nurses collegiality enhanced the possibilities for achieving the *best possible patient care*^8,10,11,24,46^ through, for example, effective communication.^11^ It was associated with increasing the quality of nursing care^11,63^ and patient safety,^15^ in terms of reduced errors^63^ and fewer adverse events.^24^

As a consequence, nurses’ collegiality *strengthened professional dignity* among nurses,^24^ including nurses their self-confidence.^11,49^ Collegiality helped to enhance nurses’ professional status^68^ and empowerment in society.^15^ It promoted how proud nurses were to be part of the nursing profession^8^ and created a bridge between different generations of nurses.^15^

Nurses’ collegiality resulted in greater *commitment to the profession*, which has been established as an important element of job satisfaction,^24,47^ motivation at work and the feeling of being seen and heard.^15^ Furthermore, collegiality was associated with better working environments,^11^ effective conflict resolution,^7,11^ lesser moral distress^15^ and staff retention.^10,11,18,47,64^

### Surrogate and related terms for the concept

According to Rodgers,^60^ surrogate terms are those that can be used instead of a concept and related terms share some attributes with the concept but do not cover the entire definition. Our analysis did not find any surrogate terms for collegiality, but the related terms were solidarity, interactions, collaboration or cooperation and collegial presence. The shared attributes with collegiality and all the related concepts were responsivity and shared responsibility for patients.^71^ However, as a concept, they did not emphasize the shared basic values and feeling of belonging that was identified in this analysis of nurses’ collegiality. As a concept, solidarity referred to the support that nurses received from each other. It was about sharing professional knowledge and skills,^72^ advocacy and promoting equality among professionals.^73^ In addition, solidarity was seen as a professional commitment to shared responsibility to healthcare, an individual or group of professionals.^74^ Nurse-to-nurse interaction referred to a professional working culture where nurses had a positive influence on each other, their interplay created a team spirit and they felt they had a responsibility to voice their concerns to colleagues if the care provided deviated from accepted practice.^75^

Collegiality has been used interchangeably with collaboration,^39^ but this is not in line with our analysis. Collaboration is a part of collegiality, and collegiality goes beyond collaboration.^4^ Collaboration has included professional collaboration, nurse-to-nurse collaboration^12^ and intra-professional collaboration.^12,71^ It has referred to the process^39^ of working together.^76^ This required joint efforts to achieve a common purpose or goal and involved individuals who have diverse, yet complimentary, skills and who use an effective communication process. In addition, collaboration has been described as a process among individuals who tend to be informally affiliated professional associates and who actively cooperate, communicate and coordinate their activities to achieve a mutually held goal. The aim of collaboration was to reach a consensus and effectively manage conflict to make sure goals were achieved. For example, one study showed that cooperation was important for a midwife’s professional wellbeing because it influenced their caring actions and their efforts to succeed.^8^ Collegiate presence was defined as a mutual connection between two or more professional individuals or groups who shared a common work focus and were mindful of cultural differences.^65^

## Discussion

Based on our analysis, the attributes of the concept of nurses’ collegiality are a professional value and a strategy to achieve shared professional goals, based on equality, reciprocity and trusted advocacy among colleagues. It is the form of autonomous self-regulation of the profession and expressed by engaged belongingness. As antecedents of the concept, collegiality requires a professional group of people who are connected to each other to make happened and those colleagues also need to share the same professional ethics and self-esteem in order to represent the profession. The consequences of the concept of nurses’ collegiality are to self-esteem and status of individuals and groups of professionals, as well as good patient care.

In the antecedents of the concept, colleagues were defined as nurses in the intra-professional sense, including only nurses with the same, or equivalent, background education, who shared work and had the same common purpose of work.^10,15,39,49,62,63,70^ These antecedents are not only partially in agreement with previous definitions but also showed differences. In previous definitions, colleagues were considered to be individuals with the same status or level of responsibility^37^ or people who knew each other.^38^ Based on our analysis, the key element and crucial *characteristics of* sameness,^37^ of nurses’ collegiality, were nurses who were seen as colleagues, regardless of what stage they were in their career^15,69^ or their professional or academic qualifications.^15,39,49,70,77^ It is noteworthy that intra-professional approaches have been reported to support the commitment that current and future generations showed to their work.^64,67^ In addition, the global community and connectedness of nurses were highlighted during the COVID-19 pandemic,^21^ and collegiality has been reconsidered also in relation to increasing common remote work. Thus, in the future, the concept of nurses’ collegiality should be studied because both the nursing profession and their working environment are constantly changing. Understanding about collegiality would support nursing staff to focus on the common purpose of work, which is providing continuous quality and safe care for patients.

Normative nature on the concept of nurses’ collegiality is identifiable. Based on our analysis, attributes as equality, reciprocity and trusted advocacy created an expectation of culture and structure,^37^ where nurses’ collegial behaviour enabled professional decisions to be made and put into practice.^39^ Collegiality is also seen as an expression of Kantian duty ethics,^37^ when a colleague has seen as an end itself and collegiality as a duty: collegial relationship provides the special reasons to realize collegiality, respect other as equals and provide relationship good in a fair and distributive manner.^23^ Thus, the normative nature of nurses’ collegiality was distinct from other related terms as collaboration, teamwork and working with others.^26^ It has also been distinguished from, and been seen to be incompatible with,^37^ congeniality or collegial friendship,^28^ connectedness and care^77^ between colleagues that were based on individual characteristics rather than of professional purposes.^37^

In this analysis, engaged belongingness was identified as an attribute of nurses’ collegiality, in line with previous studies.^37,77^ Betzler and Löschke^37^ pointed out that collegial recognition is a part of collegial relationship goods and how someone is working *toward* their colleagues. The authors stated that only colleagues could fully recognize each other’s professional skills, abilities and contributions to common work-related goals and the specific demands and pressures of a particular job. That understanding gave colleagues the authority to fully appreciate the work of their colleagues and provide them with collegial recognition.^37^ Colleagues could validate work-related experiences. Thus, the concept of nurses’ collegiality inherently has a collective element because it brought together professionals who had completing, not competing, goals. Nurses’ collegiality has been seen to have a unique meaning. It has been regarded as a desirable element in the nursing profession, which expressed a strong value basis and the desire to achieve cohesive profession. The engaged belongingness among the other attributes of collegiality can be seen as useful items in nursing management to support and encourage nurses for professional unity. Also, identified antecedents and consequences can be used for learning and developing nurses’ collegiality in clinical practice and professional education. However, future conceptual studies with empirical methods would deepen our understanding about the content of collegiality and thus to find strategies to support both individual nurses and professionals as a group to foster intra-professional collegiality.

Limiting collegiality to one profession has two sides. It is inclusive and creates loyalty among one professional group but excludes other professions. As collegiality is also connected to professional autonomy and status in society, intra-professional approaches may make the values and power of the profession to achieve good patient care more visible. Inter-professional approaches have been seen as a prerequisite to inter-professional collegiality.^62^ There is an obvious need for collaboration – and collegiality – across the professions,^78^ agencies^79,80^ and the sectors of society currently involved in healthcare settings.^81^ In future, further analysis of inter-professional collegiality would provide knowledge to support different professional stakeholders in health and social care share values, so that they can provide good care for client and patient in complex settings. Collegiality clearly is an important concept in nursing ethics, but it earns future theoretical and empirical research.

### Strengths and limitations

The strength of this study is that this was the first concept analysis on the concept of nurses’ collegiality. Based on our previous studies^15^ and in line with previous papers,^38^ we found that the terms *colleague* and *collegiality* were widely used but superficially and without definitions. These concepts were typically mentioned in the discussion sections of papers, in order to highlight the value of collegiality for the profession and for providing good care. The previous superficial use of the concept may be why concept analysis has been lacking, strengthening the need for the analysis carried out in this study. The methodological heterogeneity of the papers could be considered a limitation, but it could also be seen as a strength because we combined knowledge from different sources. To make sure that our searches were comprehensive, we used a number of databases and did not limit the search years. However, using grey literature and other professional guidelines or documents would strengthen the study. Also, limiting on the searches to papers published in English may have excluded some relevant papers.

### Research ethics

All phases of the research have been conducted with the respect of research integrity.^82^ This type of study does not need ethical approval.

## Conclusions

Nursing is value-based work that requires multidimensional collaboration with peers and other professionals. Although professional ethics creates a basis for the profession, the concept of nurses’ collegiality highlights the equal and trustful nature of achieving shared professional goals. Nurses’ collegiality is also a normative character because it creates an expectation that the nurses’ work relations will reflect shared cultures, structures and behaviour. The crucial element is that they need to share the same education and professional goals as this will create a vertical bridge over the different generations in both a profession and global sense. Collegial recognition and working in the patients’ best interest are key to that process. Thus, the concept of nurses’ collegiality has a unique meaning, as it refers to a desirable element in the nursing profession and shows that the nursing profession is strongly based on shared values and professional cohesion. It establishes the need to focus on professional self-esteem and recognition throughout all phases of nurses’ carrier. This analysis of the concept of nurses’ collegiality helps us to understand the multidimensional nature of the concepts. It also provides the opportunity for further development and application of the concept in nursing research and clinical practice.
